# Clinical validation of S100B use in management of mild head injury

**DOI:** 10.1186/1471-227X-12-13

**Published:** 2012-10-27

**Authors:** Olga Calcagnile, Linda Undén, Johan Undén

**Affiliations:** 1 Dept of Paediatric Medicine, Halmstad Regional Hospital, Halmstad, Sweden; 2 Lund University, Lund, Sweden; 3 Medical Student, Lund University, Lund, Sweden; 4 Dept of Anaesthesiology and Intensive Care, Skane University Hospital, Malmo, Sweden

## Abstract

**Background:**

Despite validated guidelines, management of mild head injury (MHI) is still associated with excessive computed tomography (CT) scanning. Reports concerning serum levels of S100B have shown promise concerning safe reduction in CT scanning but clinical validation and actual impact on patient management is unclear. In 2007, S100B was introduced into emergency department (ED) clinical management routines in Halmstad, Sweden. MHI patients with low (<0.10 mikrogram/L) levels of S100B could be discharged without CT. Our aim was to examine the clinical impact and performance of S100B in clinical use for MHI patients.

**Methods:**

Adult ([≥]18 years) patients with MHI (GCS 14–15, loss of consciousness and/or amnesia and no additional risk factors) and S100B sampling within 3 hours were prospectively included in this validation study. Patients were managed according to the adapted guidelines and management was documented. Outcome was determined with a questionnaire 3 months post-trauma and medical records to identify significant intracranial complications such as new neuroimaging, neurosurgery and/or death related to the trauma.

**Results:**

512 patients were included. 24 (4.7%) showed traumatic abnormalities on CT and 1 patient died (0.2%). 138 patients (27%) had normal S100B levels and 374 patients (73%) showed elevated S100B levels. No patients with a normal S100B level showed significant intracranial complication. 44 patients (32%) were managed with CT despite the guidelines recommending discharge (all these CT scans were normal) and 28 patients (7%) were discharged despite a CT recommendation (follow-up was normal in all these patients). S100B had a sensitivity of 100% (95% CI 83-100%) and a specificity of 28% (95% CI 24-33%) for significant intracranial complications.

**Conclusion:**

The clinical use of S100B within our existing guidelines for management of MHI is safe and effective. Adult MHI patients, without additional risk factors and with normal S100B levels within 3 hours of injury, can safely be discharged from the hospital.

## Background

Traumatic brain injuries (TBI) result in almost 17 000 emergency department (ED) visits per year in Sweden and account for more than 1 million ED visits each year in both the United States of America and the United Kingdom [1-3]. Most of them (up to 95%) are classified as mild head injuries (MHI) [4], commonly defined as a head trauma with short loss of consciousness (LOC) or amnesia for the accident, Glasgow Coma Scale (GCS) 14–15 and no neurological deficits at the time of medical inspection. These patients have been notoriously difficult to manage since they have a low, but not negligible, risk of an intracranial complication, which may be life threatening [5]. Pathological computed tomography (CT) results after MHI are found in 0.5-20% of patients (0-8% for significant complications) and the need for neurosurgical intervention is between 0-1% [6].

Scandinavian guidelines for management of minimal, mild and moderate head injuries were presented by the Scandinavian Neurotrauma Committee (SNC) in the year 2000 [1]. For patients with GCS 14–15 and LOC and/or amnesia, these guidelines recommend head CT or, as a secondary option, hospital admission with clinical observation. Similar guidelines have been published from other groups [7-9] and all have the same goal; to stratify patients with MHI into risk groups for intracranial complications. In order to ensure that guidelines do not miss patients with intracranial complications, substantial over-triage to CT has historically been accepted (between 80–99,5% of CT’s after MHI are normal [6,10]). In recent years, however, focus has been put on reducing unnecessary CT scans due to limitations in health care resources along with reports of increased cancer risks associated with exposure to medical radiation [11,12]. External comparisons of different clinical decision rules have shown favourable results for the SNC guidelines [10,13].

During the last fifteen years, protein S100B has received increasing attention as a possible biomarker for neurological disease [14,15]. Low serum levels of the protein are found in healthy individuals while patients with head trauma have a level of S100B proportionate with the severity of their brain injury [16]. S100B has a very high sensitivity for brain injuries, possibly even higher than CT [17], which would result in a high negative predictive value (NPV) in the MHI setting. Based on several studies from separate research groups and a meta-analysis [18-22], S100B has shown a NPV of over 99% for intracranial complications and close to 100% for neurosurgical lesions after MHI. Considering the theoretical CT reduction of 30%, S100B seems useful in the management of this patient group.

Despite these promising studies, S100B has not been validated in clinical practice and the impact on decision-making in a real-life setting is unclear. The aim of this study was therefore to examine the clinical impact and diagnostic performance of serum S100B levels in actual management of MHI patients.

## Methods

### Study setting and population

**Figure 1:**
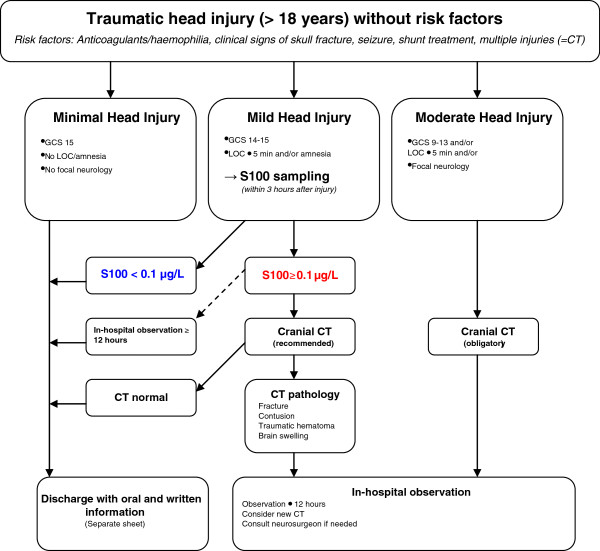
**Modified Scandinavian Neurotrauma Committee (SNC) guidelines including S100B sampling.** Dotted line indicates secondary management option. GCS = Glasgow Come Scale, CT = Computed Tomography.

In early 2007, S100B was introduced into clinical practice within the existing SNC guidelines to create new local management routines (Figure 1). The addition of S100B was applied to a group of patients, typically considered as intermediate risk for intracranial complication, where CT is normally recommended. We set the time interval for S100B sampling at 3 hours post injury, reflecting the evidence available in 2007 [23]. Also, evidence for S100B use in children at this time was relatively weak and the new guidelines were therefore used only in adults.

After a 6 months adjustment period, we undertook a prospective cohort validation study in Halmstad Regional hospital, Sweden, from November 2007 to May 2011, to evaluate the adapted guidelines explained above. Our hospital is a level II trauma centre with 24-hour emergency care, anaesthesiology, radiology, surgery and intensive care.

We consecutively enrolled all adult patients with MHI and S100B sampling. Initial inclusion criteria were therefore analogous to the MHI group in the SNC guidelines; adult patients with acute trauma to the head with GCS 14–15 during examination and loss of consciousness < 5 minutes and/or amnesia, with the addition of the S100B sample. Patients with anti-platelet agents (such as aspirin or clopidogrel) were included. Exclusion criteria were age less than 18 years, non-Swedish citizens (difficult to follow up), neurological deficits, additional risk factors from the SNC guidelines (therapeutic anticoagulation or haemophilia, clinical signs of depressed skull fracture or skull base fracture, posttraumatic seizures, shunt-treated hydrocephalus and multiple injuries) and patients where serum sampling for S100B was done more than 3 hours post-injury.

Our goal was to include 500 patients in the study, based upon consensus in the study group when considering the aim of the study. A sample size calculation was not performed.

The study was conducted in accordance to the Helsinki Declaration and approved by the Lund regional ethical committee, Lund, Sweden (reference number 19/2007). Since the study did not involve any change in patient management and based upon clinical practice, informed consent was not necessary and the ethics committee concurred with this decision.

### Blood sampling and biochemical analysis

A 5ml blood sample was drawn from each patient’s cubital vein in the ED. Samples were analysed with the fully automated Elecsys® S100 (Roche AB) at the Clinical Chemistry Department of Halmstad Regional hospital, Sweden. Roche AB report a range between 0.005 μg/L and 39 μg/L and a within-series coefficient of variance of <2.1%. Based on the available evidence at this time, we chose a cut-off level for normal levels of less than 0.10μg/L and a window of sampling of 3 hours from the time of the accident [19,23]. Lab results were available to treating physicians within 1 hour after sampling.

### CT examinations

CT scans were performed with a GE VCT Ligthspeed 64 multislice detector with a 0,625/0,625mm, 0,5 seconds rotation time and pitch of 0,531:1. 10mm thick slices were used as part of the standard CT protocol for these patients. CT scans are always analysed by a board certified radiologist and confirmed by a consultant radiologist. Since S100B was used clinically, radiologists were not blinded to S100B results. A CT scan was considered positive if any signs of cranial (skull fracture) or intracranial pathology (hematoma, air or contusion) were present.

### Standardized assessment of patients

Supervised interns and surgical residents from the ED of the Halmstad Regional Hospital assessed patients. These physicians underwent several educational sessions on evaluating patients with MHI using the new guidelines. Physicians were instructed to follow the new guidelines for all non-severe head injury patients even though deferral from these due to clinical judgement was allowed.

### Data registration and follow-up

Details of how patients were managed, including patient characteristics, type of injury, patient history, medications, clinical examination results, CT results, admission type and duration were documented in an Excel spreadsheet.

Patients were asked to answer a questionnaire sent by mail 3 months after the injury, which was repeated if no answer was received. For patients who did not return the questionnaire after these attempts, a blinded assessor conducted the questionnaire via telephone. Included in this questionnaire were questions that would identify a significant intracranial complication [7]. In cases where patients could not be reached by mail or telephone, medical records and national mortality databases were consulted for evidence of complications and/or death. Considering the rigid and transparent organisation of the health care system in Sweden, these methods would identify all patients with significant (enough to result in new neuroimaging, neurosurgery or death) intracranial complications.

Our outcome endpoint for the study was significant intracranial complication, which was defined as either a traumatic complication on emergency CT or, via follow-up, new neuroimaging showing traumatic intracranial complication or neurosurgery and/or death due to an intracranial complication.

Sensitivity, specificity, positive and negative predictive values were estimated from cross tabulation between S100B and significant intracranial complications and reported with corresponding 95% confidence intervals. Values are reported to two significant figures.

## Results

**Figure 2:**
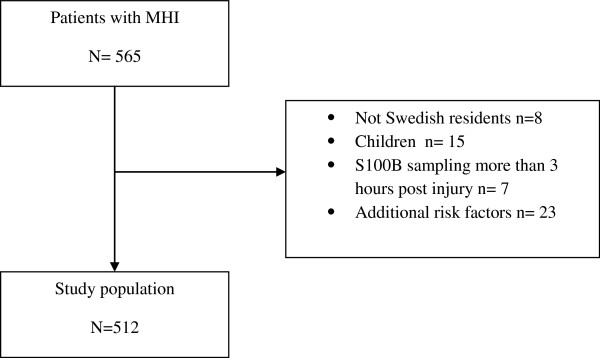
**Inclusion process.** MHI = Mild Head Injury.

Between November 2007 and May 2011, we enrolled 512 patients (see Figure 2 for inclusion process and Table 1 for descriptive statistics). 26 patients had cranial CT pathology but only 24 (4.7%) showed traumatic abnormalities (isolated skull fracture n=3, cerebral contusions n=7, acute subdural hematoma n=3, intracranial air n=1, combinations of traumatic intracranial findings n=10). 2 patients showed CT pathology not related to trauma (cerebral tumour n=1 and pathological intracranial calcification n=1). No patients needed neurosurgical intervention. One patient died as a result of a head injury; an 83-year-old man with an S100B level of 0.23μg/L and a CT showing expansive cerebral contusions who died from increased intracranial pressure. Neurosurgical care was denied due to advanced age.

**Table 1:** Descriptive statistics

	**S100B < 0.10μg/L**	**S100B ≥ 0.10μg/L**	**All**
Male	85 (61.6%)	229 (61.3%)	314 (61.5%)
Female	53 (38.4%)	145 (38.7%)	198 (38.5%)
Age (mean)	32.6	46.6	42.2
Total	138	374	512

**Figure 3:**
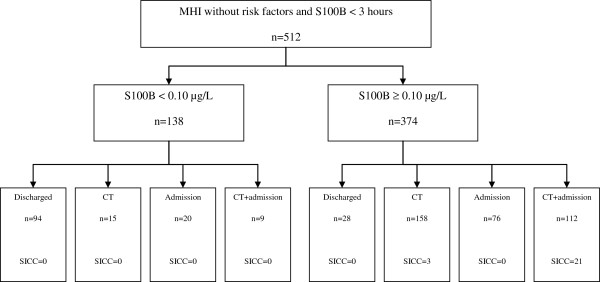
**Patient management in the study cohort including number of intracranial injuries.** CT= computed tomography, MHI= mild head injury, SICC=Significant Intracranial Complication.

138 patients (27%) had a S100B level less than 0.10μg/L and 374 patients (73%) showed a S100B level higher or equal to 0.10μg/L. Details of how patients were managed are presented in Figure 3. The follow up questionnaire was completed for 414 patients (81%). Medical records and the mortality database were successfully checked for all remaining patients. No patients with a normal S100B level showed significant intracranial complication, either on CT or on follow-up, see Figure 3.

Over-triage (CT or admission performed when the guidelines recommended discharge) occurred in 44 patients (32%) with normal S100B levels. 15 of these had a CT scan, 20 were admitted and 9 patients had both a CT and admission. All of these patients had normal CT findings and/or normal follow-up. Under-triage (not performing a CT when recommended) occurred in 28 patients (7%) with elevated S100B levels. None of these patients had any significant intracranial complications on follow-up.

S100B displayed a sensitivity and NPV of 100% for significant intracranial complications, a specificity of 28% and a positive predictive value (PPV) of 6%, see Table 2.

**Table 2:** Cross tabulation showing statistical values for S100B and significant intracranial complications

	**SICC + Total = 24**	**SICC - Total = 488**	
**S100B ≥ 0.10μg/L**	24	350	**PPV**: 6,4%
Total = 374			(95% CI 4.2-10%)
**S100B < 0.10μg/L**	0	138	**NPV**: 100%
Total = 138			(95% CI 97-100%)
	**Sensitivity**: 100%	**Specificity**: 28%	
	(95% CI 83-100%)	(95% CI 24-33%)	

SICC = Significant intracranial complications, NPV= Negative predictive value, PPV= Positive predictive value.

## Discussion

The first report concerning serum S100B as a possible biomarker in MHI was published in 1995 [15]. Since then, numerous reports and a meta-analysis, documenting the potential of S100B to safely reduce CT scans following MHI, have increased the evidence for clinical use [20-24]. However, actual clinical validation has never been reported despite the biomarker being used clinically in several European countries. In 2007, S100B was introduced as a clinical tool in the management of MHI in our hospital, in an attempt to reduce CT scans after these injuries. This study shows that this implementation has been successful and that S100B, using a cut-off of less than 0.10 μg/L for normal values and a time window of 3 hours from injury, shows similar predictive values to the derivation studies.

Low compliance to guidelines is a common problem [5]. 32% of patients with normal S100B levels were over-triaged with CT, admission or both. None of these had any intracranial complications. It is natural to expect caution when using new routines, especially concerning an injury where biomarkers have never been used before. Also, physicians must always be free to exert clinical judgement since management guidelines are merely an aid in the clinical process. Some patients cannot be sent home from the ED irrespective of S100B and/or CT findings (for example; elderly patients without support in their home environment, serious intoxication and patients with other injuries).

Our adapted guidelines are based upon the evidence-based SNC management guidelines from the year 2000 [1]. Since this publication, considerable new evidence has emerged in this field, including validated guidelines based upon patient history and clinical examination [7-9]. The impact of including S100B in other guidelines is unknown. However, the SNC guidelines have proved accurate in comparison studies [8,10] so the implementation of S100B into these is justifiable. Despite this, the examination of S100B within other guidelines is naturally warranted.

Owing to the predictive properties of S100B, the biomarker is best adapted into an intermediate risk group of patients, such as in this study. The prevalence of traumatic intracranial injury in this group was 4.7%, similar to other cohorts. These patients would normally receive a CT recommendation according to the SNC guidelines, which is justifiable considering the prevalence level. However, interpreting S100B levels in minimal head injury would lead to substantial over-triage (false positives) and using levels in more severe head injuries could lead to under-triage and may risk missing important complications (false negatives) [12].

This study has several limitations. Firstly, one may argue that our method of determining the outcome measure, significant intracranial complications, may miss patients that may in fact have CT abnormalities. However, if these exist, these abnormalities would not have resulted in any change in management and/or outcome for these patients. The organisation of the state-owned Swedish health care system, with personal identification numbers connected with all medical journals, allows us to accurately identify new neuroimaging, neurosurgery and/or death in all patients who were not followed up with the questionnaire and therefore identifies any cases of important intracranial injury. This also allows us to minimise recall bias arising from the questionnaire. Secondly, none of our patients needed neurosurgery. If this was the endpoint, one could suggest that all our patients could have been discharged without S100B or CT. This management, however, would not be accepted in Sweden and the results must be considered in relation to the existing guidelines, which recommend CT in all these patients, similar to guidelines in other countries. Thirdly, the timing of S100B sampling after injury may be of importance. We used a time window of 3 hours based upon the evidence at this time [23] and worries concerning the short half-life of S100B in blood [25]. Recently, a large prospective study has utilised a time window of 6 hours [20] with maintained predictive ability of S100B. It seems reasonable that a time window of 6 hours may be more applicable to this population and should be considered in future studies and/or clinical practice. Finally, deviation from the guidelines was seen. Although this was allowed in the study protocol, reasons for the deviation were not explored in depth and would have been an interesting point to examine. Future studies should include a comparison of clinical rules with unstructured physician assessment, in order to fully explore this aspect, including reasons for deviation from a guideline.

## Conclusion

Incorporation of S100B into existing guidelines for management of MHI in adults is safe and effective. Adult MHI patients without additional risk factors and with normal S100B levels within 3 hours of injury can safely be discharged from the hospital.

## Competing interests

The authors declare that they have no competing interests.

## Authors’ contributions

JU conceived and designed the study, with input from LU and OC. JU, OC and LU acquired data. OC and JU did the analysis and interpretation of data. OC drafted the manuscript, JU and LU did critical revision and all authors finally approved the manuscript.

## Pre-publication history

The pre-publication history for this paper can be accessed here:

http://www.biomedcentral.com/1471-227X/12/13/prepub
